# The effect of ethanol evaporation on the properties of inkjet produced liposomes

**DOI:** 10.1007/s40199-020-00340-1

**Published:** 2020-04-18

**Authors:** Ruba Bnyan, Laura Cesarini, Iftikhar Khan, Matt Roberts, Touraj Ehtezazi

**Affiliations:** 1 School of Pharmacy and Biomolecular Sciences, Liverpool John Moores University, L3 3AF, Liverpool, UK; 2 School of Pharmacy, University of Camerino, 62032 Camerino, MC Italy

**Keywords:** Inkjet method, Liposome, Nano- size, PDI, Ethanol content, Rotary evaporator

## Abstract

**Background:**

Inkjet method has been used to produce nano-sized liposomes with a uniform size distribution. However, following the production of liposomes by inkjet method, the solvent residue in the product could have a significant effect on the properties of the final liposomes.

**Objective:**

This research paper aimed to find a suitable method to remove ethanol content and to study its effect on the properties of the final liposomal suspension.

**Method:**

Egg phosphatidylcholine and lidocaine were dissolved in ethanol; and inkjet method at 80 kHz was applied to produce uniform droplets, which were deposited in an aqueous solution to form liposomes. Dry nitrogen gas flow, air-drying, and rotary evaporator were tested to remove the ethanol content. Liposome properties such as size, polydispersity index (PDI), and charge were screened before and after ethanol evaporation.

**Results:**

Only rotary evaporator (at constant speed and room temperature for 2 h) removed all of the ethanol content, with a final drug entrapment efficiency (EE) of 29.44 ± 6.77%. This was higher than a conventional method. Furthermore, removing ethanol led to liposome size reduction from approximately 200 nm to less than 100 nm in most samples. Additionally, this increased the liposomal net charge, which contributed to maintain the uniform and narrow size distribution of liposomes.

**Conclusion:**

Nano-sized liposomes were produced with a narrow PDI and higher EE compared to a conventional method by using an inkjet method. Moreover, rotary evaporator for 2 h reduced effectively the ethanol content, while maintaining the narrow size distribution.

**Figure:**
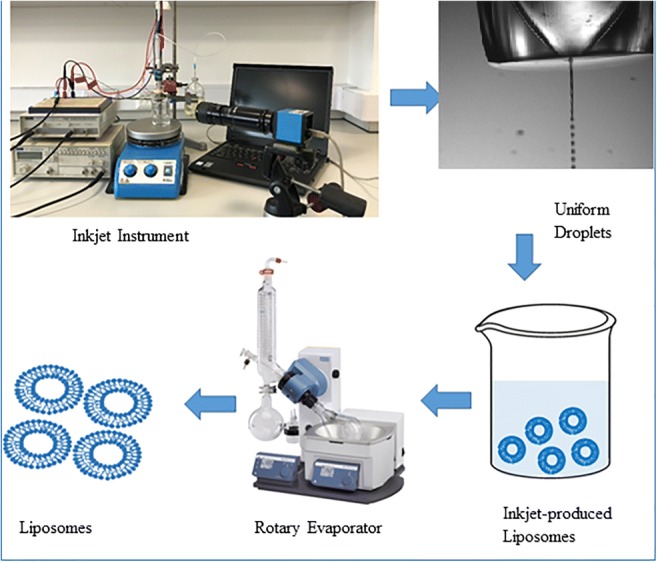
Graphical abstract

## Introduction

Since their invention in the 1960s [1], liposomes have been used in the delivery of a wide range of therapeutic agents such as methotrexate [2] and nucleic acid [3]. Novel approaches have been taken to increase the therapeutic efficacy of liposomes [4]. For example, drug loaded liposomes have been covalently attached to multi-walled carbon nanotubes (CNT) to form CNT-liposome conjugates [5]. This approach allowed uptake of liposomes by human embryonic kidney (HEK) 293 cells, where unconjugated liposomes failed to enter the cells [5]. Moreover, the surface of liposomes were decorated with nanodiamond (ND) nanoparticles. The ND nanoparticles adsorbed onto the liposomes via hydrogen bonding. The presence of ND nanoparticles at the surface of liposomes facilitated their uptake, while bare liposomes could not be internalised by HeLa cells [6]. In addition, it is important to control the size and size distribution of liposomes as the cellular uptake of liposomes is size dependent also; and even the mechanism of uptake changes with size [7]. For example liposomes with sizes of 97.8 nm and 162.1 nm were subjected to clathrin-dependent uptake, while smaller liposomes (40.6 nm) primarily followed a dynamin-dependent pathway by Caco-2 cells [7].

Relatively small liposomes were prepared with an average of 100 nm and a polydispersity ndex (PDI) of 0.25 by the extrusion method utilising polycarbonate membranes (with pore size in the range of 100–400 nm) [8]. To produce uniform nanoparticles, microfluidic methods [9–15], or coaxial turbulent jet mixing have been employed [16]. Microfluidic techniques produced liposomes in the range of 80–90 nm with PDI of 0.11–0.22 [11], unilamellar liposomes in the range of 30–40 nm with PDIs as small as 0.11 [12], or liposomes with sizes as small as 27 nm [15]. Furthermore, a combination of microfluidics and Design-of-Experiment allowed the preparation of up to 30 liposome formulations a day. The optimised liposomes had sizes of 109.3 ± 15.3 nm with PDIs less than 0.25 [17]. To scale-up the production of uniform nanoparticles by microfluidic methods, a coaxial turbulent jet mixer was developed to produce lipid vesicles with sizes of 100 nm at a production rate of 3 kg/d [16]. These studies show the need for the production of nanoparticles with sizes less than 100 nm with a narrow size distribution.

Inkjet method produces uniform droplets from an inkjet device [18], and this technique has been used to produce uniform liposomes with a size in the range of 20–100 nm [19], uniform respirable particles for the formulation of pharmaceutical inhalers [20, 21], and uniform porous polymer particles [22]. For the production of liposomes, the amphiphilic compounds were dissolved in ethanol and printed into an aqueous solution [19]. Inkjet method also have been employed to produce uniform unilamellar lipid vesicles. In this approach, the drug solution was transformed into a jet of uniform droplets by an inkjet device. Then, each droplet hit the surface of a solution containing lipid bilayer membrane at liquid/air interface. Each lipid vesicle was formed by the sequence of membrane deformation, membrane collapse and vesicle separation [23].

Following the production of liposomes either by applying microfluidic techniques or inkjet methods, the solvent residue in the product could have safety concerns [9]. Hence, purification is required to remove excess surfactants, uncapsulated drug and residual of organic solvents [10, 15]. However, it remains to be investigated, whether the purification method itself could affect the loading degree or particle size distribution of liposomes. A previous study applied a supercritical extraction method to remove the ethanol residue from liposomes, which were formed by dropwise addition of ethanol solution containing phosphatidylcholine into water by a stainless steel needle. Applying the supercritical fluid reduced the size of liposomes from 358 nm to 164 nm [24]. Therefore, this paper aimed to prepare uniform small liposomes by inkjet method and applying an alternative method of purification such as rotary evaporation to remove solvent residues without affecting liposomal properties. Lidocaine was chosen as model of a low molecular weight active ingredient. Lidocaine loaded liposomes were reported many times in literature [25–29]. Hence, a conventional method for the production of liposomes was investigated in this study to compare both methods. The effects of purification method were evaluated on the size, size distribution and the morphology of inkjet-produced liposomes.

## Materials and method

### Materials

Egg phosphatidylcholine (EPC) was purchased from Sigma Aldrich, UK. Lidocaine (97.5%), acetonitrile (ACN), methanol, Tween 80, and absolute ethanol were obtained from Fisher Scientific, UK. Di-Potassium hydrogen orthophosphate anhydrous was purchased from BDH Chemicals Ltd., UK. All solvents used were of HPLC grade. Formvar film 200 mesh cupper grids were purchased from Agar scientific, UK. Spectra/Pro®3 dialysis membrane with molecular weight cut-off of 10 kDa was purchased from Fisher Scientific, UK. Polytetrafluoroethylene (PTFE) in-Line filters with 0.2 μm pore size were purchased from VWR (UK).

### Method

#### Preparation of lipid solution for inkjet method

Several lipid solutions were freshly prepared (Table 1). Blank lipid solution was prepared by dissolving 500 mg of EPC in 100 ml of ethanol solution. Additionally, a drug-lipid solution was also prepared, where 150 mg of lidocaine was dissolved with 500 mg of EPC in 100 ml of ethanol. A drug loaded liposome formulation with half lipid concentration was also produced.

**Table 1:** Summary of the prepared formulations, showing the components and the amounts of both reservoir and receiver solutions

Samples (Abbreviation)		Reservoir solution		Receiver solution
		EPC lipid (mg)	Lidocaine (mg)	component
Blank liposome 1	(BL1)	500	–	Distilled Water
Loaded liposome 2	(LL2)	500	150	Distilled Water
Blank liposome 3	(BL3)	500	–	Tween 80 solution
Loaded liposome 4	(LL4)	500	150	Tween 80 solution
Loaded liposome 5	(LL5)	250	150	Distilled Water

The amounts of EPC lipid and lidocaine are given in 100 ml of ethanol

#### Liposome preparation by inkjet method

**Fig. 1:**
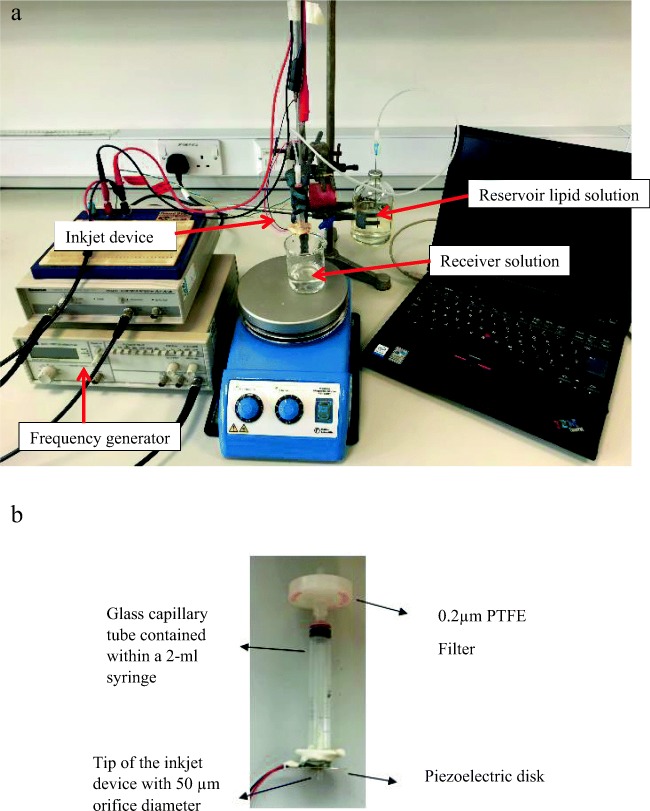
Image of the in-house inkjet instrument: (**a**) a typical assembly of the in-house inkjet apparatus, (**b**) close-up image of inkjet device showing the piezoelectric disk and the nozzle tip

Blank liposomes and lidocaine-loaded liposomes were produced using an inkjet instrument designed in-house (Fig. 1). Ehtezazi et al. have reported the instrument design and setup in previous papers [20, 21]. Briefly, the inkjet instrument was built using a glass capillary tube, which was already adjusted at one end to form a nozzle orifice of a selected size (50 μm and 20 μm). The tip of the nozzle was also supplied with a piezoelectric disk to transfer the ultrasonic waves to the solution at this point. The glass capillary tube was accommodated inside a 2-ml syringe for the ease of handling and forming connections. The syringe luer was connected to a PTFE membrane in-Line filter. The lipid solution was fed to the filter by a plastic tube connected to a 100-ml reservoir filled with the lipid solution. When it was suspected that the filter was shedding particles into the inkjet device (caused nozzle blockage), the reservoir was cleaned with particle free water, and after drying the lipid solution was filtered into the reservoir bottle using PTFE filter. The height of the reservoir bottle was adjusted (lowered compared to the tip of the nozzle) to prevent solution dripping from the nozzle, but at the same time ensuring the presence of the lipid solution at the tip of the nozzle. This arrangement was essential to ensure that particles (if they existed) in the reservoir would not find their ways to the inkjet nozzle. The uniform droplets produced by the inkjet device were delivered to a receiver container with distilled water or surfactant (Tween 80) solution. The Inkjet-produced droplets travelled for 7 cm before hitting the surface of the solution in the receiver container. The inkjet device was actuated at 80 kHz using a TG315; Thurlby Thander Instruments function generator, which was connected to an amplifier (Thurlby Thander Instruments). An amplitude of 20.8 V was used. The droplets were visualised by using a 10× objective lens (Mitutoyo, Japan), CCD camera (EC1020; Prosilica, Vancouver, British Columbia, Canada) and telescope (Navitar, Rochester, New York) [19]. The surfactant solution was prepared at a concentration of 0.007 mg/ml using Tween 80. A constant volume of 5 ml was used in the receiver container; and typically 2.5 ml of lipid solution was added to the receiver by the inkjet device. Additionally, continuous stirring was applied in the receiver container using a magnetic stirrer at the speed of 150 rpm at room temperature to rapidly disperse the droplets (produced by the inkjet device) deposited into the receiver solution during the whole time of droplet production by the inkjet device.

#### Liposome preparation by conventional method

Conventional liposomes were prepared using the traditional thin film hydration method [30], where 500 mg of EPC and 150 mg of lidocaine were dissolved in 100 ml of ethanol and sonicated for 5 min. After that, the ethanol was evaporated using a rotary evaporator (Heidolph Laborota 4000 efficient, Germany). A thin lipid film was formed after complete evaporation at 250 rpm rotation. The thin film was then hydrated with 5 ml of water and left to anneal for 30 min. The obtained liposomes were then subjected to sonication for 1 min using probe sonicator (QSonica sonicators, USA) at 40% amplitude in ice-bath.

#### Ethanol evaporation

Three methods were investigated to evaporate ethanol content from the liposomes by the inkjet method: keeping the liposome solution in an open petri dish (air-drying) [31], exposing the liposome solution to dry nitrogen gas [32], and using a rotary evaporator [33, 34]. Rotary evaporator (Heidolph Laborota 4000 efficient, Germany) was used at 250 rpm at room temperature under reduced pressure. Dry nitrogen gas led to erratic outcomes, and air-drying caused the formation of drug crystals. Hence, rotary evaporation was mainly considered for evaporating the ethanol. However, optimisation of the evaporation time was also required. The ethanol content of the liposome solution was determined after 90 and 120 min of evaporation using the gas chromatography method explained below.

#### Gas chromatography with flame ionisation detection for ethanol quantification

An Agilent Technologies® 6890 N Network Gas Chromatography (GC) system equipped with Flame ionisation detection was used to quantify ethanol content in the samples of interest using nitrogen gas as a mobile phase with a flow rate of 3.2 ml/min. A calibration curve was prepared using five different concentrations of ethanol by dilution of the specified volume of ethanol i.e. 0.2, 0.4, 0.6, 0.8, 1.0 ml into 10 ml of water. All experiments were conducted in triplicates.

#### Size analysis and zeta potential measurement

Both the conventional liposomes and all produced batches of inkjet liposomes (Table 1) were analysed for size, PDI and zeta potential before and after ethanol evaporation. One ml of each batch of liposomes was placed into a transparent cuvette and analysed at room temperature (25 °C) in a dynamic light scattering (DLS) instrument (Zetasizer Nano; Malvern Instruments Ltd., UK).

#### Liposome loading efficiency

The encapsulation efficiency (EE) of both the inkjet-produced- liposomes and the conventional liposomes were obtained by measuring the unentrapped lidocaine concentration using an in-house developed HPLC method [33]. Then the EE was calculated using the following equation:

$$ \%\mathbf{EE}=\frac{\left(\mathbf{total}\ \mathbf{drug}\ \mathbf{conc}.-\mathbf{unentrapped}\ \mathbf{drug}\ \mathbf{conc}.\right)}{\mathbf{Total}\ \mathbf{drug}\ \mathbf{conc}.}\times \mathbf{100} $$

#### Liposome morphology

Liposome morphology was checked using both transmission electron microscopy (TEM) and scanning electron microscopy (SEM). For TEM a drop of liposome solution was applied to Formvar® coated copper grids (Agar Scientific, UK) and left to dry for few minutes. The sample was visualised using a FEI Morgagni Transmission Electron Microscope (Philips Electron Optics BV, Netherlands). For SEM, the sample was pipetted onto microslide and left to dry overnight. The dried samples were gold coated using a Emitech K550® coater and visualised with a Philips XL20® Scanning electron microscope.

#### In vitro release study

The release profile of liposomes was carried out using a strip of dialysis bag with cut-off size of 10 kDa for samples prepared by the inkjet method. The inkjet device was operated for 20 min and the droplets were deposited into a 50 ml beaker containing 5 ml of distilled water as explained in the above. One end of the bag was sealed, 3.5 ml of the sample was pipetted into the bag, and then the opposite end was sealed as well to trap the sample within the bag. The bag was fully immersed into a 50-ml conical flask containing 36.5 ml of distilled water. The flask was left on a magnetic stirrer set at 150 rpm and room temperature to ensure mixing of release media. Aliquots of 0.5 ml were sampled from the external media at different time intervals: 0, 1, 2, 3, 5, 7, and 24 h and replaced with a fresh 0.5 ml of distilled water. The aliquots were then analysed by HPLC [33] to check the lidocaine content.

## Results and discussion

### Results

#### Ethanol evaporation attributes

**Fig. 2:**
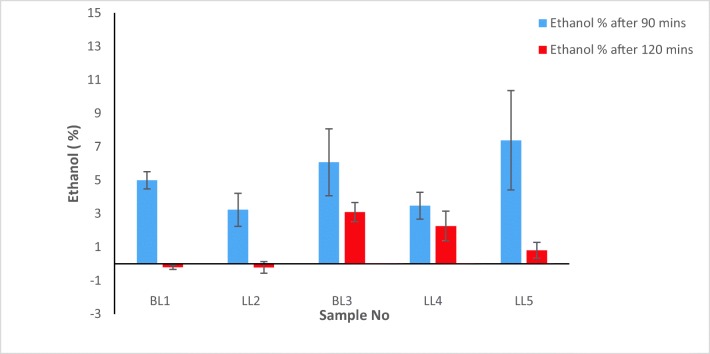
The ethanol percentage in each formulation after using the rotary evaporator for 90 min (blue bars) or after 120 min (red bars), *n* = 3 and error bars are showing ±SD

Three methods for ethanol evaporation have been tested in this study, which include leaving the inkjet-produced samples in open-air petri dish, using direct flow of nitrogen gas into the sample, and using a rotary evaporator. However, preliminary observations indicated that the first two methods were ineffective. Upon using nitrogen gas flow led to the whole solution evaporation, while leaving the samples for 24 h in petri dish led to drug precipitation or crystallisation. Therefore, only the results from rotary evaporation are reported here. Ethanol content of the produced samples were then measured after 90 or 120 min of rotary evaporation. Ethanol percentage in all samples were reduced to less than 10% after 90 min of rotary evaporation, whereas a further reduction to almost 0% for ethanol was found following an additional 30 min of evaporation process (Fig. 2).

#### Size, PDI and charge analysis

**Fig. 3:**
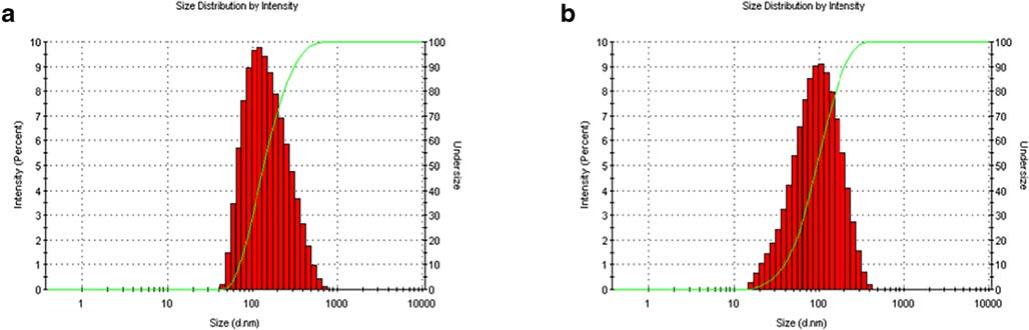
Illustration of the obtained size distribution of sample LL2; (**a**) size distribution before ethanol evaporation, (**b**) size distribution after ethanol evaporation

In this work an inkjet device with the orifice size of 20 μm was also used to produce uniform liposomes with the aim of achieving smaller liposomes. However, the liposomes produced by the 20-μm inkjet device were similar to liposomes produced by the 50-μm inkjet device, apart from longer duration time (eight times more) of inkjet process. Therefore, the results of 50-μm inkjet device are presented here. The results of the inkjet-produced liposome size, PDI, and charge before and after ethanol evaporation are shown in Table 2. The formed liposomes in all samples had sizes below 200 nm before evaporation except BL1 that had liposome size of 347.73 nm (Table 2). However, after ethanol evaporation the sizes of all produced liposomes decreased to less than 100 nm, whereas BL1 decreased to less than 200 nm. Generally, all samples showed good PDI around 0.2 before and after removing ethanol except BL3 and LL4 samples that showed higher PDI after removing ethanol. Considering the loaded samples only, LL2 was the sample that showed the smallest liposome size and the best PDI before and after ethanol evaporation (Fig. 3). On the other hand, the obtained conventional liposomes had size of 412 ± 8.35 nm before sonication with PDI of 0.88 ± 0.02, however the size reduced to 361 ± 8.46 nm after sonication and the PDI was 0.534 ± 0.05. Additionally, the conventional liposomes charge was −8.90 ± 0.25 mV. Inkjet-produced liposomes also had similar surface charges (Table 2).

**Table 2:** Size and charge analysis results of all inkjet-produced formulations. Mean values ± SD, *n* = 3

Sample	Before evaporation			After 120 min Evaporation		
	Size ± SD (nm)	PDI	Zeta potential (mV)	Size ± SD (nm)	PDI	Zeta potential (mV)
BL1	347.73 ± 51.36	0.10 ± 0.04	−6.24 ± 1.05	183.30 ± 37.16	0.20 ± 0.04	−16.96 ± 0.57
LL2	137.23 ± 4.81	0.21 ± 0.01	−13.66 ± 1.56	63.55 ± 4.07	0.24 ± 0.01	−38.26 ± 0.12
BL3	90.81 ± 46.24	0.25 ± 0.02	−3.39 ± 0.92	64.92 ± 4.31	0.45 ± 0.09	−10.56 ± 0.75
LL4	156.56 ± 34.14	0.22 ± 0.01	−17.53 ± 0.98	87.32 ± 20.43	0.34 ± 0.06	−37.96 ± 2.43
LL5	178.30 ± 12.63	0.18 ± 0.03	−10.30 ± 0.32	88.82 ± 3.99	0.18 ± 0.03	−37.40 ± 2.57

#### Encapsulation efficiency

The encapsulation efficiency of the loaded inkjet sample was evaluated after removing the ethanol content. The inkjet sample (LL2) showed encapsulation efficiency of 29.44 ± 6.77%, while the encapsulation efficiency of the conventional liposome preparation was 7.81 ± 1.22%.

#### Liposome morphology

**Fig. 4:**
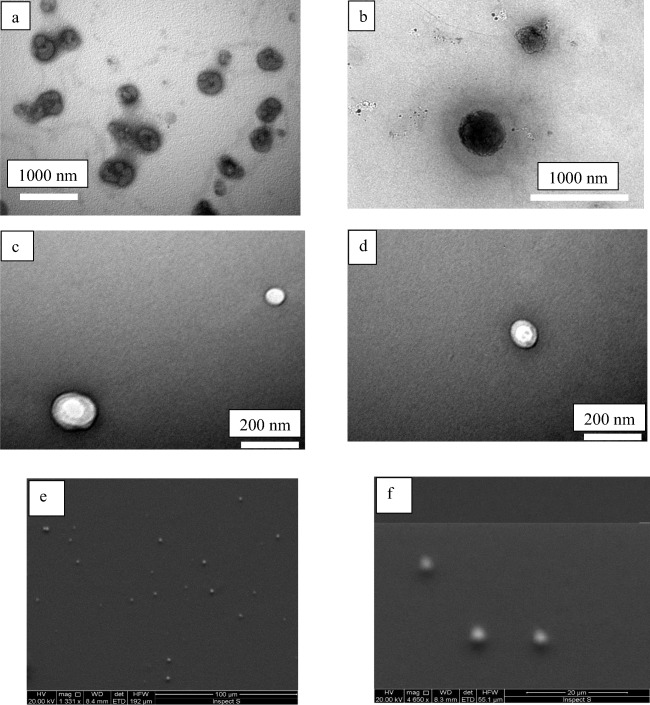
**a** and **b** TEM images of inkjet-produced liposomes before ethanol evaporation, and (**c** and **d)** TEM images of inkjet-produced liposomes after ethanol evaporation (90 min). **e** and **f** SEM images of inkjet-produced liposomes prior to removing ethanol confirming the spherical and uniform shape of liposomes

The morphology of drug-loaded inkjet-produced liposomes was observed using both SEM and TEM (Fig. 4). TEM images show the liposomes before and after (90 min) ethanol evaporation. It is evident from the images that inkjet method produced liposomes with spherical shape and intact spherical bilayer membrane. SEM images present the formation of uniform liposomes by the inkjet method. These were obtained in preliminary studies, while attempts were made to observe the liposomes for formulation of.

#### In-vitro release results

As LL2 formulation showed suitable drug entrapment and lowest ethanol content following rotary evaporation, this formulation was chosen for the release studies. Hence, five replicates were produced. After 20 min of producing droplets by the inkjet method for LL2 formulation, the batch volume was 8.5 ± 0.4 ml (*n* = 5). As each batch contained 5 ml of distilled water and the inkjet device was operated at 80 kHz (80,000 droplets per second), the calculated droplet diameter becomes approximately 42 μm (taking into account 4% volume reduction when water and alcohol are mixed). This is less than the inkjet device orifice diameter (50 μm). As the inkjet method produces droplets comparable to the orifice diameter in the drop-on demand mode, then part of the ethanol should have evaporated during the inkjet process (either when the droplets were in the air or/and in the receiver aqueous solution while mixing). Assuming 50 μm for each ethanol droplet produced by the inkjet method, the total ethanol volume becomes 6.28 ml delivered by the inkjet device within 20 min.

**Fig. 5:**
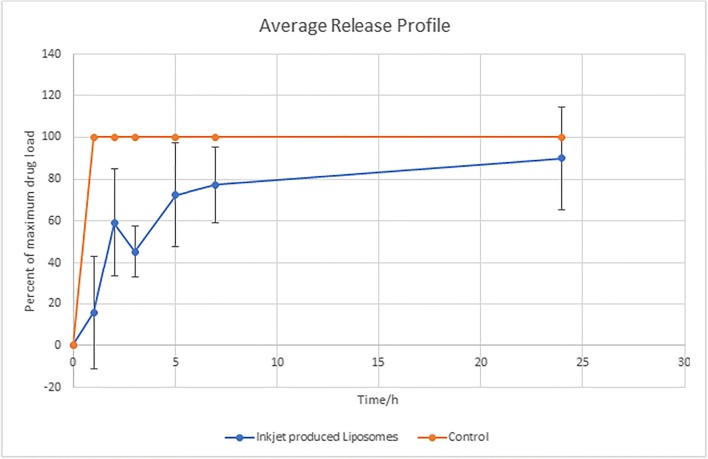
Release profiles of lidocaine from ink-produced liposomes (LL2 formulation) versus the control (free drug). Error bars indicate standard deviation (*n* = 3)

The release profiles were obtained for three replicates of inkjet-produced liposomes (LL2 formulation) versus the release of control (free drug alone). Free drug showed almost 100% release of the drug within the first hour while the inkjet-produced liposomes slowly released the drug over the studied time with 45.39 ± 12.40% release (of maximum drug load in the dialysis bag at time t = 0 h) after 3 h (Fig. 5). As it can be seen from Fig. 5, there was a significant variation between replicates at the early hours of drug release study. This suggested variable burst release of un-encapsulated lidocaine in liposomes. The total drug released was 5.33 ± 1.98 mg. The batch residual volume was 6.3 ± 1.3 ml after 2 h of rotary evaporation.

### Discussion

Liposomes with a controlled size and narrow size distribution were successfully produced using an inkjet method in this study. All liposome sizes were below 200 nm before evaporation (Table 2), similar to previously reported literature [19]. However, the main challenge was to find a method to remove or reduce the organic solvent (ethanol) from the liposome solution without altering the liposomal size or PDI. It has been reported that presence of ethanol have an effect on the product stability and safety as it could cause the bilayer to be leaky, tend to increase the liposome size, reduce the entrapment efficiency as well as solubilise the vesicles if present at a very high percentage [35–37].

Using a constant speed rotary evaporator at room temperature was proven an efficient method to remove a high percentage of the ethanol content (Fig. 2). Evaporation for an hour and 30 min reduced the ethanol content to approximately 4% in most samples, except LL5. Additionally, further evaporation for another 30 min successfully removed all of the ethanol content, except for the inkjet-produced samples that receiver solution contained Tween 80. Both blank and drug-loaded inkjet-produced samples that the receiver solution contained Tween 80, showed 3% and 1% of ethanol content even after 2 h of evaporation. It was suggested that high concentrations of ethanol could causes interpenetration of the solvent within the hydrocarbon chain, which could also be enhanced by the presence of surfactant [38]. This could be the explanation of some samples retaining more ethanol after evaporation in comparison to others.

It was reported that 40% ethanol would reduce liposome size by 45% in comparison with non-ethanol based liposomes [19]. Moreover, others suggested that increasing the ethanol content would decrease the liposome size [35, 38]. Conversely, this study has demonstrated that liposomes have been produced with generally very small size (< 200 nm), and narrow PDI in comparison to other reported methods of liposome production (Table 2, before evaporation). Additionally, reducing ethanol content in most samples had a positive effect on the size reduction. There was a noticeable size reduction after using the rotary evaporator for 2 h (Table 2, after evaporation), with all samples showing a reduction of approximately 100 nm whilst retaining similar PDI. In contrast, conventional liposomes that were prepared using the most common reported method of liposome preparation, thin film hydration method, they showed larger size even after probe sonicating them using ultrasound sonicator. These liposomes showed a wider PDI, which was around 0.5 in comparison with the inkjet samples. Moreover, evaporating ethanol had a noticeable effect on the liposomal solution zeta potential. The charge has increased dramatically in all samples (Table 2), as it ranged from -3nmV to −17.53 mV before evaporation to −10 mV and − 37.96 mV after evaporation of samples BL3 and LL4 respectively. It has been previously proposed that ethanol content has an effect on the net charge and stability of the system [39–43]. In addition, this observation may be explained by the fact that the zeta potential of liposomes is directly related to the dielectric constant of the solvent [44]. As dielectric constant of water is greater than that for ethanol [45], then removing ethanol from the liposome solution would increase the dielectric constant of media, and consequently the zeta potential. A high net charge after ethanol evaporation could increase the degree of steric stabilisation and hence decrease the liposome size [38, 43].

Moreover, using the inkjet method proved to enhance the entrapment efficiency of liposomes in comparison with the conventional method of preparation. Encapsulation efficiency of 22–36% of hydrophobic drug is considered acceptable as the drug could be placed within the lipid bilayer [46]. Low encapsulation efficiency of hydrophobic drug was reported in literature; and it was suggested that the small size cannot offer enough space within the lipid bilayer to load more drug [30, 33]. However, the inkjet method enhanced the drug encapsulation in comparison to the conventional method, where EE% of 8% was achieved with the latter method. This could be explained by the burst of liposomes during the probe sonication, while the inkjet method saved the liposomes from being ruptured during the preparation. This may be supported by observing images of both TEM and SEM before and after ethanol evaporation (Fig. 4), which showed spherical shape and intact bilayer membrane of the inkjet liposomes with uniform size. Therefore, the ethanol evaporation did not affect the inkjet-produced liposomes. Additionally, the release profile of the inkjet liposomes proved that the liposomes were able to release the drug slowly with 45% release achieved after 3 h without showing a major burst release or the need to be tailored and have a coat to sustain the drug release as reported previously [47–49]. However, the error bars were larger compared to previously reported work, in particular for the early hours of drug release profile [50]. One explanation could be due to the mechanical stresses that were imposed on the liposomes during the release studies by the magnetic bar. Hence further investigations are required to identify appropriate dialysis method for these liposomes [50]. Finally, the rotary evaporation process itself should be monitored and standardised to avoid large residual batch volume variations.

## Conclusion

Nano-sized liposomes with a narrow size distribution and encapsulation efficiency of about 30% have been successfully produced using an inkjet method. Also, the inkjet method showed to be more efficient in producing drug-loaded and nano-sized liposomes in comparison to the most common thin film hydration method of producing liposomes. This study has tested the effects of the ethanol evaporation on the properties of inkjet-produced liposome. It was found that using a rotary evaporator at constant speed at room temperature for 2 h would effectively remove all of the ethanol content. Furthermore, it was proven that removing ethanol resulted in liposomal size reduction and increased liposomal net charge, which in turn helped maintaining the uniform size distribution due to the repulsion effect between the liposomes.
